# Comminuted Trapezium Fracture: Case Presentation and Review of Surgical Fixation Techniques

**DOI:** 10.1155/2021/5532713

**Published:** 2021-05-27

**Authors:** Amanda F. Spielman, Sriram Sankaranarayanan

**Affiliations:** ^1^Miller School of Medicine, University of Miami, 1600, NW 10th Avenue, Miami, Florida, USA 33136; ^2^Department of Orthopedic Surgery, NYU Langone Health, Woodhull Hospital Center, New York City, New York, USA 11206

## Abstract

We report a case of a 28-year-old male who sustained a comminuted trapezium fracture with carpometacarpal subluxation of the right hand. Treatment with internal fixation with a headless compression screw resulted in excellent outcomes.

## 1. Introduction

Trapezium fractures are rare injuries occurring in less than 4 percent of all hand fractures [1–5]. The most common types of trapezium fractures include body and ridge avulsion fractures. Body fractures can be a vertical split or a comminuted fracture.

The trapezium is palpable dorsally at the base of the thumb or distal portion of the snuffbox as well as at the base of the thenar eminence. The most common presentation of fracture is pain and tenderness at the base of the first metacarpal bone and snuffbox [6]. Patients may present with pain with wrist flexion with resistance due to the position of the flexor carpal radialis [7]. The trapezium is an important component aiding in the versatile range of motion of the thumb.

Early detection of trapezium fractures can help prevent permanent impairment such as chronic pain, joint stiffness, and reduced grip strength [4, 5]. There are limited reports of trapezium fractures and no consensus on the management of these fractures. While nondisplaced fractures may be treated with immobilization, displaced fractures often require surgical intervention [8].

We present a case of a 28-year-old male who sustained a comminuted trapezium fracture with carpometacarpal subluxation of the right hand.

### 1.1. Statement of Institutional Review Board

As no patient information was divulged in the presentation of the case, no IRB approval was required per our institutional policy.

## 2. Case Presentation

**Figure 1:**
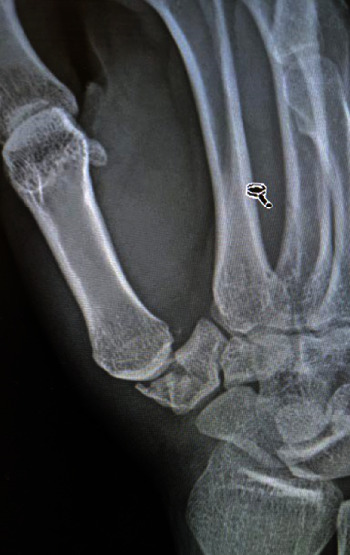
Plan radiograph at presentation demonstrating trapezium fracture with associated carpometacarpal subluxation.

**Figure 2:**
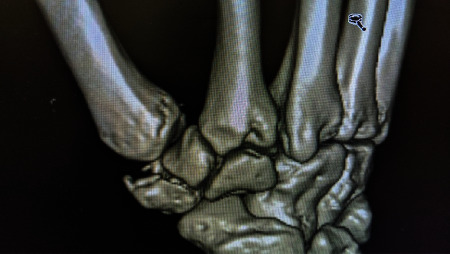
CT with 3D reconstruction detailing trapezium fracture.

A 28-year-old male with no significant medical history presented to our emergency room five days after sustaining a right-hand injury from a fall on an outstretched hand while playing basketball. On examination, there was swelling and tenderness on palpation at the base of the right thumb. Right hand radiographs (Figure 1) and CT with 3D reconstruction (Figure 2) showed a comminuted trapezium fracture.

**Figure 3:**
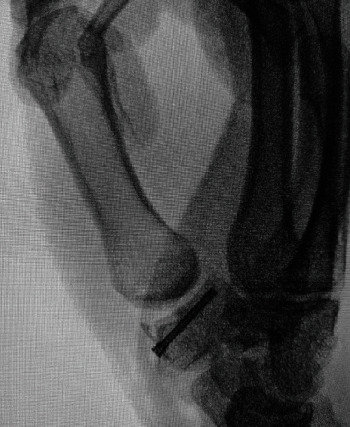
Fixation with 2 mm headless screw demonstrating excellent fixation of fracture and congruity of the carpometacarpal articulation.

The patient was taken to the operating room seven days after his initial injury. A dorsal radial approach was used to expose the trapezium protecting the radial sensory nerves, radial arteries and EPL and EPB tendons. After exposing the trapezium, we reduced the fracture with a dental pick and provisional Kirschner wires. C arm was utilized throughout the procedure to confirm fracture and joint reduction as well as to appropriately size the screw. A 2 mm headless compression screw (HCS, Synthes Inc, West Chester, PA, USA) was utilized to definitively fix the fracture (Figure 3).

Intraoperatively, there was excellent stability of the fracture and restoration of the articular surface.

**Figure 4:**
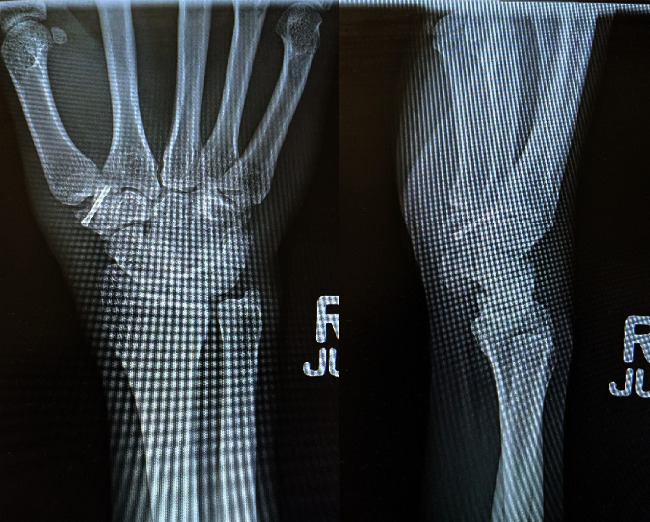
At 3 months follow-up, healing of the fracture demonstrated.

The patient's joint was immobilized with a thumb spica splint for two weeks. Unrestricted range of motion exercises as well as occupational therapy were initiated two weeks following surgery. Three months after surgery, repeated X-rays showed a healed trapezium fracture (Figure 4). During this time, his DASH score was 10 and his SANE score [9] was 95.

**Figure 5:**
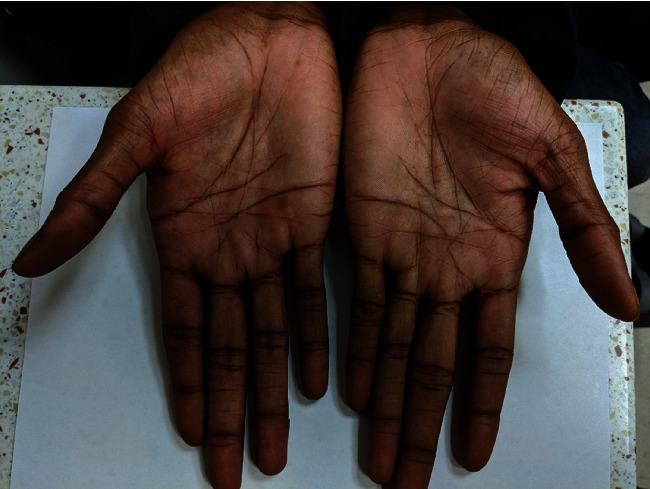
At 3 months follow-up, full range of motion of thumb demonstrated.

The patient demonstrated full range of motion of the thumb (Figure 5) and returned to all previous activities.

## 3. Discussion

Trapezium fractures are rare among hand fractures. A high degree of suspicion for the injuries aids in their diagnosis. AP wrist radiographs can identify these fractures. A pronated view can allow for better visualization of the trapezio metacarpal articulation and identify displacements. However, there are reports of missed trapezium fractures in the literature, and a CT scan can be obtained in case of a suspected trapezium fracture. A CT scan delineates the fracture pattern and aids in surgical planning [3]. Magnetic resonance imaging (MRI) is an option to identify occult trapezium fractures and bone bruising; however, it is used infrequently [10].

The most common types of trapezium fracture include a vertical split pattern or a comminuted pattern. Often there may be an overlap between these two types. The Walker Classification describes five types of trapezium fractures based on pattern and joint involvement: I—horizontal, IIa—radial tuberosity through CMC joint, IIb—radial tuberosity though scaphotrapezial joint, III—ulnar tuberosity, IV—vertical, and V—comminuted fracture [11]. Trapezium fractures may be seen in association with Bennet's fracture of the metacarpal [7].

While there is a paucity of literature on the appropriate management of trapezium fractures, a few case series have reported different treatment options for these injuries including nonoperative management. Authors have advocated different surgical techniques for management of comminuted trapezium fractures [12–14]. The different techniques include the use of K wires, cross pinning of the first metacarpal to the second metacarpal, headless compression screws, or plates for fracture fixation. Bone grafts have been used for greater defects. These case reports have demonstrated good to excellent outcomes with almost all patients regaining pain-free and useful range of motion [7, 11, 15–17]. While there are a few reports on the use of K wire for fixation of these fractures, there is no clear consensus exists on whether open or closed pinning is preferred.

Our case describes a patient with a comminuted trapezium fracture with carpometacarpal subluxation. We hypothesize that the comminuted trapezium fracture was caused by an axial loading of the trapezium by the first metacarpal during the fall. A 3D CT analysis allowed for visualization of the complex fracture and aided in surgical planning. As there was significant displacement of the fracture along with a carpometacarpal subluxation, a decision was made to perform an open reduction and internal fixation of the fracture. We elected to use a headless compression screw over K wires as we believed that we would be able to begin early range of motion in our patient with the use of headless compression screw.

At follow-up, the patient demonstrated excellent outcomes and use of his thumb. His DASH score was 10, and his SANE score was 95. The Single Assessment Numeric Evaluation (SANE) is a patient reported single outcome score used to evaluate a patient's rating of current function of the affected hand in relation to normal. The assessment is a single patient-reported value from 0–100 (100 being normal function) [9]. The SANE score has been validated as a quick and useful tool to obtain patient outcomes in hand surgery [9]. The patient had a near normal return of his hand function as seen by his self-reported SANE score of 95 as well as his DASH score.

The novelty of this case report lies in that trapezium fractures are rare injuries of the hand. There are no clear guidelines on the best management strategy for these fractures. We believed that an adequate restoration of the articular congruity of the trapezium was important for the long-term hand function in a young patient, such as the one presented in our case report. Hence, we elected to perform an open reduction and internal fixation of the trapezium fracture with a headless compression screw. The case report demonstrates the excellent outcome that can be obtained with this approach.

## 4. Conclusion

Trapezium fractures are a rare injury of the hand with no clear consensus in the literature with regard to their management. We report a case of a patient with a comminuted trapezium fracture who underwent an open reduction and internal fixation with a headless compression screw with excellent outcomes.
